# Association between marital status and all-cause mortality of patients with metastatic breast cancer: a population-based study

**DOI:** 10.1038/s41598-023-36139-8

**Published:** 2023-06-05

**Authors:** Shouqiang Zhu, Chong Lei

**Affiliations:** Department of Anesthesiology and Perioperative Medicine, Xijing Hospital, The Fourth Military Medical University, Xi’an, 710032 China

**Keywords:** Cancer, Psychology, Oncology, Risk factors

## Abstract

This study aimed to investigate the association between marital status and the prognosis of patients with metastatic breast cancer (MBC). Data of patients with MBC were obtained from the Surveillance, Epidemiology, and End Results (SEER) database. Patients were classified into married and unmarried groups. Kaplan–Meier analysis with the log-rank test was conducted to compare breast cancer-specific survival (BCSS) and overall survival (OS) between the groups. Univariable and multivariable Cox proportional models were used to determine whether marital status was independently associated with OS, and the Fine–Gray subdistribution hazard method was performed to determine whether marital status was independently associated with BCSS. In total, 16,513 patients with MBC were identified, including 8949 married (54.19%) and 7564 unmarried (45.81%) patients. The married patients were significantly younger [median age (interquartile range), 59.0 (50.0–68.0) vs. 63.0 (53.0–75.0); p < 0.001] and received more aggressive treatments, such as chemotherapy (p < 0.001) and surgery (p < 0.001), than the unmarried patients. Moreover, married patients had higher 5-year BCSS (42.64% vs. 33.17%, p < 0.0001) and OS (32.22% vs. 21.44%, p < 0.0001) rates. Multivariable analysis revealed that marital status was an independent prognostic factor, and married status was associated with a significant reduction in the risk of breast cancer-specific (sub-hazard ratio, 0.845; 95% confidence interval, 0.804–0.888; p < 0.001) and all-cause (hazard ratio, 0.810; 95% confidence interval, 0.777–0.844; p < 0.001) mortality. Unmarried patients had a 15.5% increased risk of breast cancer-specific mortality and a 19.0% increased risk of overall mortality compared with married patients with MBC. BCSS and OS were superior in married populations compared with unmarried populations in most subgroups. Marital status was an independent prognostic indicator for survival in patients with MBC and was associated with significant survival benefits.

## Introduction

Breast cancer is a common malignancy and is the leading cause of cancer-related mortality in women. Approximately 297,790 new cases of invasive breast cancer and 55,720 cases of female breast ductal carcinoma in situ will be diagnosed in the United States in 2023, accounting for 31% of all new cancer cases in women^1^. Although advances in early detection and treatment have improved survival rates, the incidences of breast cancer metastasis and recurrence remain high. Approximately 6% of patients present with metastatic breast cancer (MBC) at their initial diagnosis^2^. Metastasis is an independent predictor of death from breast cancer^3,4^.

Psychosocial factors have been shown to significantly affect tumorigenesis and prognosis, with marital status being a crucial factor^5,6^. Extensive research has demonstrated that married individuals tend to exhibit healthier lifestyles, including regular physical activity, healthy diet, and frequent medical checkups, which may serve as intermediary factors for cancer prevention^7^. Marital status is strongly correlated with the prognosis of various malignancies^8–10^. Married individuals tend to have better access to emotional and financial support and more comprehensive medical care, and thus have a better prognosis^11–13^. Previous studies have investigated the impact of marital status on breast cancer survival outcomes, demonstrating that unmarried patients are more likely to be diagnosed at an advanced stage and experience greater mortality across all American Joint Committee on Cancer stages compared with married patients^25–27^. However, these studies did not include patients with molecular typing or first diagnosis of stage IV breast cancer. Therefore, whether marriage offers greater benefits to patients with MBC remains unclear. To address these gaps, the present study aimed to investigate the association between marital status and the prognosis of patients with MBC.

## Methods

### Availability of data and materials

The present study used data sourced from the National Cancer Institute-supported Surveillance, Epidemiology, and End Result (SEER) program (https://seer.cancer.gov/), which encompasses 18 distinct population-based cancer registries, accounting for approximately 32% of the United States population^14^. A SEER Research Data Agreement (reference number: 18124-Nov 2020) was duly executed to obtain authorization for data extraction in strict compliance with the approved research procedures.

### Population

The SEER database is an assemblage of regional population-based cancer registries that document patient demographics, tumor characteristics, cancer-related treatment, and mortality information^14^. In this study, we used SEER*Stat 8.3.9.2 software to identify eligible patients from the incidence-SEER 18 Registries Custom Data, including additional treatment fields, and investigated the association between marital status and survival outcomes in patients with MBC. The study data were collected from January 2010 to December 2015, and patients who met the inclusion criteria, such as those diagnosed with breast cancer (ICD-O-3 codes 8500-8599) and primary breast cancer, were included. Patients aged < 18 years, those with multiple primary tumors, those diagnosed with MBC via autopsy and death certificate, or those without metastasis were excluded. The final analysis only included records with complete information on marital status and survival outcomes.

### Variables

This study investigated several variables related to breast cancer, including age at diagnosis, race, surgical intervention, chemotherapy, radiation, tumor grade, breast cancer subtype, metastasis, and marital status. Age was analyzed as a continuous variable using the median (quartile), and subgroup analyses were performed to compare those aged > 65 years with those aged < 65 years. Race was categorized into four groups: White, Black, other (Asian/Pacific Islander and American Indian/Alaska Native), and unknown. Breast surgery was classified into five categories: no surgery, partial mastectomy, simple mastectomy, radical mastectomy, and unknown. Chemotherapy and radiotherapy were defined as yes, no, or unknown. The pathological tumor grade was divided into five groups: well-differentiated (grade I), moderately differentiated (grade II), poorly differentiated (grade III), undifferentiated/anaplastic (grade IV), and unknown. Molecular subtyping of breast cancer was based on hormone receptor (HR) and human epidermal growth factor receptor 2 (HER2) expression: HR (–) and HER 2 (–), HR (–) and HER 2 ( +), HR ( +) and HER 2 (–), HR ( +) and HER 2 ( +), and unknown. The HR status of the tumor was stratified into HR-positive (ER + /PR + , ER–/PR + , and ER + /PR–) and HR-negative (ER–/PR–). Distant organ metastases (limited to the bone, lungs, brain, and liver in this study) were classified into four groups: none, one site, multiple sites, and unknown. Marital status was dichotomized into married and unmarried, including separated, single, widowed, divorced, or domestic partner, and was collected through patient self-report or from information provided by family members or healthcare providers. We evaluated breast-cancer-specific survival (BCSS) and overall survival (OS). BCSS was defined as the duration between diagnosis and death specifically attributed to breast cancer, whereas OS was calculated as the time from diagnosis to death from any cause.

### Statistical analysis

A two-sample *t* test was used to compare normally distributed continuous variables between binary groups; otherwise, the Mann–Whitney *U* test was performed. The normality of the variables was evaluated using the Kolmogorov–Smirnov test. Chi-squared or Fisher’s exact test was used for categorical variables. No more than 15% missing values were recorded for all variables (Supplementary Fig. 1). This study aimed to investigate the effect of marital status on the OS and BCSS of patients with MBC. To address the issue of missing data in several covariates, such as race, tumor subtype, and distant organ metastasis, we opted not to exclude patients with incomplete data. Instead, we used an “unknown” dummy variable to account for the absence of data. To assess the association between marital status and survival rates, we used the Kaplan–Meier method with log-rank tests. We further utilized univariable and multivariable Cox proportional hazards models to identify independent prognostic variables for OS. We used the Fine and Gray’s test to evaluate the difference in BCSS rates between groups and the Fine–Gray subdistribution hazard method to identify independent predictors.

To explore the potential effect of various covariates on the association between marital status and survival, we conducted subgroup analyses based on age, race, distant organ metastasis, chemotherapy, surgery, tumor grade, and subtype. For the cause of death analysis, we classified patients with an unknown cause of death as dying of a non-tumor-related cause in the primary analysis, whereas in the subsequent sensitivity analysis, we classified these patients as dying of a tumor-specific cause. Moreover, we employed competing-risk models based on the Fine and Gray method to account for the competitive relationship between non-tumor and tumor deaths.

We included all patients who completed the follow-up period, and no data regarding the duration of follow-up were missing. To evaluate the robustness of our findings, we conducted a sensitivity analysis by excluding patients with missing covariate data and those who survived for < 1 month after diagnosis. Finally, we conducted a causal mediation analysis to explore the possible mechanisms underlying the association between marital status and better outcomes. We used chemotherapy and surgery as mediator variables, marital status as an independent variable, and long-term survival as a dependent variable. To perform these mediation analyses, we used the R package *mediation*, which implements causal mediation analysis using both parametric and non-parametric methods. This approach allowed us to estimate the total effect of the independent variable on the dependent variable, as well as the direct and indirect effects through the mediators, while controlling for potential confounding variables. All statistical analyses and visualizations were performed using SPSS (version 26.0; SPSS Inc., Chicago, USA), R (version 4.1.2; R Foundation for Statistical Computing, Vienna, Austria), and Stata (version 14.0; Stata Corp., College Station, TX, USA). Statistical significance was defined as a two-sided p-value of < 0.05.

### Ethics committee

We completed the SEER project registration and obtained authorization for data extraction (reference number: 18124-Nov 2020). Data extraction strictly followed the research procedures of the approved protocols. Because the database is publicly available and data were de-identified, approval from an ethics committee is not required.

## Results

### Characteristics of patients with MBC

**Figure 1:**
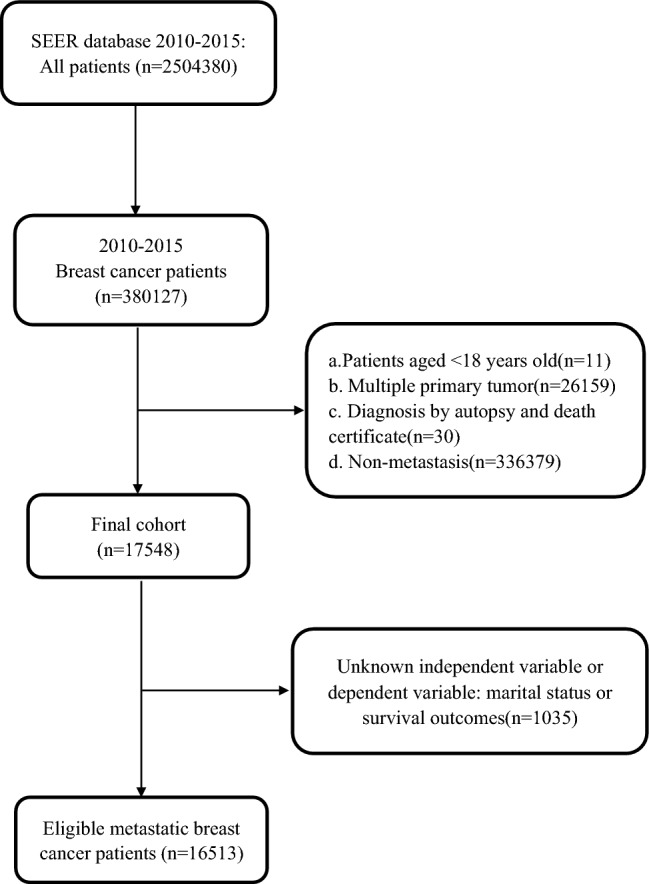
Flowchart describing eligible patients from the SEER database.

A total of 38,0127 patients were diagnosed with breast cancer as their first primary malignancy between 2010 and 2015, of which 16,513 were eligible for inclusion in the study of MBC. The final analysis, presented in Fig. 1, comprised 7564 (45.81%) married and 8949 (54.19%) unmarried patients. White ethnicity, younger age, and receipt of chemotherapy and surgery were more prevalent among married patients. However, no significant differences were found in the pathological tumor grade (p = 0.234). Statistically significant differences were observed in radiation and distant organ metastases; however, the clinical significance of these differences was minimal. Table 1 provides a detailed summary of the patient characteristics stratified by marital status.

**Table 1:** Baseline characteristics.

Characteristics	Total (n = 16,513)	Unmarried group (n = 8949)	Married group (n = 7564)	p
Age (yr), median (IQR)	61.0 (52.0–71.0)	63.0 (53.0–75.0)	59.0 (50.0–68.0)	< 0.001
Race, n%				< 0.001
White	12,407 (75.13)	6346 (70.91)	6061 (80.13)	
Black	2764 (16.74)	2001 (22.36)	763 (10.09)	
Other	1296 (7.85)	576 (6.44)	720 (9.52)	
Unknown	46 (0.28)	26 (0.29)	20 (0.26)	
Grade, n%				0.234
I	1170 (7.09)	631 (7.05)	539 (7.13)	
II	6017 (36.44)	3245 (36.26)	2772 (36.65)	
III	6755 (40.91)	3626 (40.52)	3129 (41.37)	
IV	99 (0.60)	54 (0.60)	45 (0.59)	
Unknown	2472 (14.97)	1393 (15.57)	1079 (14.26)	
Tumor subtype, n%				< 0.001
HR(–) & HER2(–)	2018 (12.22)	1115 (12.46)	903 (11.94)	
HR(–) & HER2( +)	1306 (7.91)	677 (7.57)	629 (8.32)	
HR( +) & HER2(–)	9201 (55.72)	4958 (55.40)	4243 (56.09)	
HR( +) & HER2( +)	2489 (15.07)	1311 (14.65)	1178 (15.57)	
Unknown	1499 (9.08)	888 (9.92)	611 (8.08)	
Chemotherapy, n%				< 0.001
No	7465 (45.21)	4489 (50.16)	2976 (39.34)	
Yes	9048 (54.79)	4460 (49.84)	4588 (60.66)	
Radiation, n%				< 0.001
No	10,937 (66.23)	6052 (67.63)	4885 (64.58)	
Yes	5207 (31.53)	2695 (30.12)	2512 (33.21)	
Unknown	369 (2.23)	202 (2.26)	167 (2.21)	
Surgery, n%				< 0.001
No surgery	10,951 (66.32)	6211 (69.40)	4740 (62.67)	
Partial mastectomy	1554 (9.41)	750 (8.38)	804 (10.63)	
Simple mastectomy	1308 (7.92)	645 (7.21)	663 (8.77)	
Radical mastectomy	2542 (15.39)	1260 (14.08)	1282 (16.95)	
Unknown	158 (0.96)	83 (0.93)	75 (0.99)	
Distant organ metastasis, n%				< 0.001
No	1990 (12.05)	1000 (11.17)	990 (13.09)	
One site	9374 (56.77)	5069 (56.64)	4305 (56.91)	
Multiple site	4959 (30.03)	2773 (30.99)	2186 (28.90)	
Unknown	190 (1.15)	107 (1.20)	83 (1.10)	

Distant organ metastasis only included liver, brain, bone, and lung metastases in the SEER database (2010–2015).

### Marital status and MBC survival

**Figure 2:**
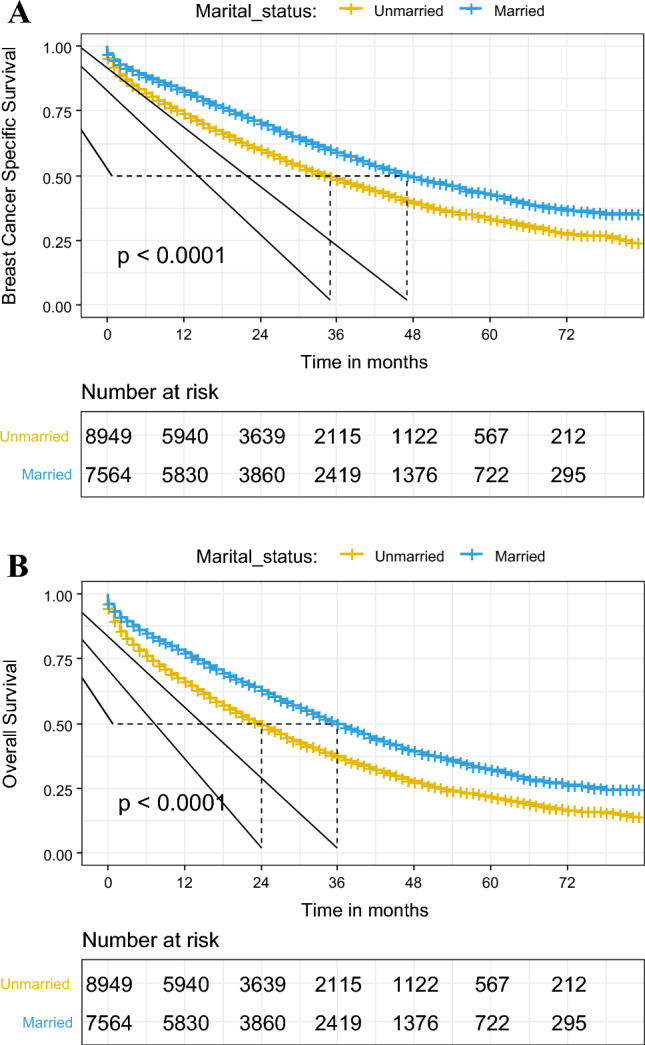
Kaplan–Meier survival curves of breast cancer-specific survival (**A**) and overall survival (**B**) in different marital statuses.

Differences in both BCSS and OS were observed between married and unmarried patients (p < 0.0001 for both, based on the log-rank test), with married patients exhibiting superior outcomes, as demonstrated in the Kaplan–Meier curve (Fig. 2). The five-year BCSS and OS rates were 42.64% and 32.22% for married patients, and 33.17% and 21.44% for unmarried patients, respectively. Cox regression and competing risk models showed that marital status was a significant prognostic factor for OS and BCSS, even after adjusting for various covariates (Table 2). In particular, in the multivariable Cox models adjusted for a list of sequentially added covariates (including age, race, grade, tumor subtype, chemotherapy, radiation, surgery, and distant organ metastases), marital status remained an independent prognostic factor for both BCSS (sub-hazard ratio, 0.845; 95% confidence interval, 0.804–0.888; p < 0.001) and OS (hazard ratio, 0.810; 95% confidence interval, 0.777–0.844; p < 0.001) (Table 3), with better outcomes observed in married patients compared with unmarried patients.

**Table 2:** Gray’s test and Fine–Gray proportional subdistribution hazard method analysis of breast cancer-specific survival (BCSS) and multivariable cox regression analyses of overall survival (OS) for patients with metastatic breast cancer.

Characteristic (n)	Univariable analysis		Multivariable analysis	
	Gray’s test	*p-*value	SHR (95% CI)	*p-*value
BCSS				
Marital status	113.425	< 0.001		< 0.001
Unmarried (8949)			Reference	
Married (7564)			0.845 (0.804–0.888)	
Ages	15.267	< 0.001		0.551
< 65 (9811)			Reference	
≥ 65 (6702)			1.016 (0.964–1.072)	
Race	74.555	< 0.001		< 0.001
White (12,407)			Reference	
Black (2764)			1.164 (1.092–1.242)	< 0.001
Other (1296)			0.979 (0.893–1.073)	0.647
Unknown (46)			0.421 (0.194–0.914)	0.029
Grade	248.417	< 0.001		< 0.001
I (1170)			Reference	
II (6017)			1.332 (1.195–1.485)	< 0.001
III (6755)			1.798 (1.610–2.009)	< 0.001
IV (99)			2.029 (1.500–2.745)	< 0.001
Unknown (2472)			1.479 (1.311–1.668)	< 0.001
Subtype	620.687	< 0.001		< 0.001
HR(–) & HER2(–) (2018)			Reference	
HR(–) & HER2( +) (1306)			0.499 (0.447–0.557)	< 0.001
HR( +) & HER2(–) (9201)			0.493 (0.455–0.533)	< 0.001
HR( +) & HER2( +) (2489)			0.416 (0.379–0.456)	< 0.001
Unknown (1499)			0.561 (0.503–0.627)	< 0.001
Chemotherapy	79.255	< 0.001		< 0.001
No (7465)			Reference	
Yes (9048)			0.845 (0.798–0.895)	
Radiation	17.511	< 0.001		NI
No (10,937)				
Yes (5207)				
Unknown (369)				
Surgery	440.224	< 0.001		< 0.001
No surgery (10,951)			Reference	
Partial mastectomy (1554)			0.634 (0.579–0.694)	< 0.001
Simple mastectomy (1308)			0.587 (0.531–0.649)	< 0.001
Radical mastectomy (2542)			0.740 (0.691–0.793)	< 0.001
Unknown (158)			1.025 (0.826–1.273)	0.820
Distant organ metastasis	685.312	< 0.001		< 0.001
No (1990)			Reference	
One site (9374)			1.249 (1.149–1.358)	< 0.001
Multiple sites (4959)			1.927 (1.763–2.105)	< 0.001
Unknown (190)			1.331 (1.057–1.677)	0.015

OS	HR (95% CI)	*p*-value	HR (95% CI)	*p*-value
Marital status		< 0.001		< 0.001
Unmarried (8494)	Reference		Reference	
Married (7564)	0.695 (0.668–0.723)		0.810(0.777–0.844)	
Ages		< 0.001		< 0.001
< 65 (9811)	Reference		Reference	
≥ 65 (6702)	1.514 (1.456–1.575)		1.360 (1.304–1.418)	
Race		< 0.001		< 0.001
White (12,407)	Reference		Reference	
Black (2764)	1.318 (1.253–1.386)	< 0.001	1.236 (1.174–1.302)	< 0.001
Other (1296)	0.930 (0.861–1.004)	0.064	0.986 (0.912–1.065)	0.717
Unknown (46)	0.459 (0.254–0.828)	0.010	0.408 (0.226–0.736)	0.003
Grade		< 0.001		< 0.001
I (1170)	Reference		Reference	
II (6017)	1.253 (1.146–1.370)	< 0.001	1.275 (1.166–1.394)	< 0.001
III (6755)	1.664 (1.525–1.816)	< 0.001	1.722 (1.573–1.885)	< 0.001
IV (99)	1.790 (1.398–2.290)	< 0.001	2.008 (1.567–2.573)	< 0.001
Unknown (2472)	1.723 (1.567–1.896)	< 0.001	1.493 (1.355–1.644)	< 0.001
Subtype		< 0.001		< 0.001
HR(–) & HER2(–) (2018)	Reference		Reference	
HR(–) & HER2( +) (1306)	0.412 (0.377–0.450)	< 0.001	0.404 (0.370–0.442)	< 0.001
HR( +) & HER2(–) (9201)	0.420 (0.397–0.444)	< 0.001	0.356 (0.335–0.379)	< 0.001
HR( +) & HER2( +) (2489)	0.332 (0.308–0.358)	< 0.001	0.308 (0.285–0.332)	< 0.001
Unknown (1499)	0.638 (0.591–0.689)	< 0.001	0.503 (0.464–0.546)	< 0.001
Chemotherapy		< 0.001		< 0.001
No (7465)	Reference		Reference	
Yes (9048)	0.664 (0.638–0.690)		0.642 (0.614–0.672)	
Radiation		< 0.001		0.005
No (10,937)	Reference			
Yes (5207)	0.813 (0.779–0.849)	< 0.001	0.946 (0.905–0.988)	0.012
Unknown (369)	0.633 (0.545–0.736)	< 0.001	0.834 (0.718–0.970)	0.018
Surgery		< 0.001		< 0.001
No surgery (10,951)	Reference		Reference	
Partial mastectomy (1554)	0.543 (0.504–0.585)	< 0.001	0.586 (0.543–0.633)	< 0.001
Simple mastectomy (1308)	0.522 (0.481–0.566)	< 0.001	0.565 (0.521–0.614)	< 0.001
Radical mastectomy (2542)	0.560 (0.528–0.594)	< 0.001	0.640 (0.602–0.681)	< 0.001
Unknown (158)	0.840 (0.688–1.026)	0.087	0.842 (0.689–1.028)	0.091
Distant organ metastasis		< 0.001		< 0.001
No (1990)	Reference		Reference	
One site (9374)	1.135 (1.062–1.213)	< 0.001	1.122 (1.050–1.200)	0.001
Multiple sites (4959)	1.924 (1.796–2.061)	< 0.001	1.860 (1.733–1.995)	< 0.001
Unknown (190)	1.543 (1.291–1.845)	< 0.001	1.373 (1.148–1.643)	0.001

Distant organ metastasis only included bone, liver, lung, and brain metastases in the SEER database (2010–2015). Patients with an unknown cause of death were classified as dying of a non-tumor-related cause. There were 7389 deaths from tumors, 2614 deaths from other causes, and 6510 survivors at the follow-up endpoint.

*NI:* The competing risk analysis in Stata software was performed using the “stcrreg” command, where NI refers to the variable being excluded from the multivariable analysis model. *SHR* subdistribution hazard ratio.

**Table 3:** Multivariable survival analysis of breast cancer-specific survival and overall survival in metastatic breast cancer.

OS	Model 1 HR (95% CI)	Model 2 HR (95% CI)	Model 3 HR (95% CI)	Model 4 HR (95% CI)	Model 5 HR (95% CI)
Unmarried	Reference	Reference	Reference	Reference	Reference
Married^†^	0.695(0.668–0.723)*	0.784(0.752–0.817)*	0.778(0.747–0.811)*	0.808(0.775–0.842)*	0.810(0.777–0.844)*
Married^‡^	0.697(0.665–0.732)*	0.779(0.741–0.818)*	0.762(0.725–0.800)*	0.794(0.755–0.834)*	0.794(0.756–0.835)*

BCSS	SHR (95% CI)	SHR (95% CI)	SHR (95% CI)	SHR (95% CI)	SHR (95% CI)
Married^†^	0.791(0.755–0.830)*	0.831(0.791–0.873)*	0.824(0.784–0.866)*	0.849(0.808–0.893)*	0.845(0.804–0.888)*
Married^‡^ 1	0.789(0.753–0.827)*	0.826(0.787–0.867)*	0.818(0.779–0.860)*	0.844(0.803–0.887)*	0.840(0.799–0.883)*
Married^‡^ 2	0.784(0.742–0.827)*	0.821(0.776–0.868)*	0.806(0.762–0.853)*	0.837(0.790–0.886)*	0.837(0.790–0.887)*

Model 1: unadjusted.

Model 2: adjusted for age and race.

Model 3: adjusted for age, race, grade, and tumor subtype.

Model 4: adjusted for age, race, grade, tumor subtype, chemotherapy, radiation (only in overall survival analysis), and surgery.

Model 5: adjusted for age, race, grade, tumor subtype, chemotherapy, radiation (only in overall survival analysis), surgery, and distant organ metastasis.

The adjusted age is a continuous variable. *SHR* subdistribution hazard ratio.

*p < 0.001.

^†^Multivariable survival analysis with metastatic breast cancer.

^‡^Sensitivity analysis of breast cancer-specific survival in metastatic breast cancer by excluding all patients with missing covariate data (n = 3744) and those who survived less than 1 month (n = 792) after diagnosis.

^‡^1: Sensitivity analysis of breast cancer-specific survival in metastatic breast cancer through classified patients with unknown cause of death as tumor-specific death.

^‡^2: Sensitivity analysis of breast cancer-specific survival in metastatic breast cancer by excluding all patients with missing covariate data (n = 3744), those who survived less than 1 month (n = 792) after diagnosis with an unknown cause of death.

### Sensitivity and subgroup analyses

**Figure 3:**
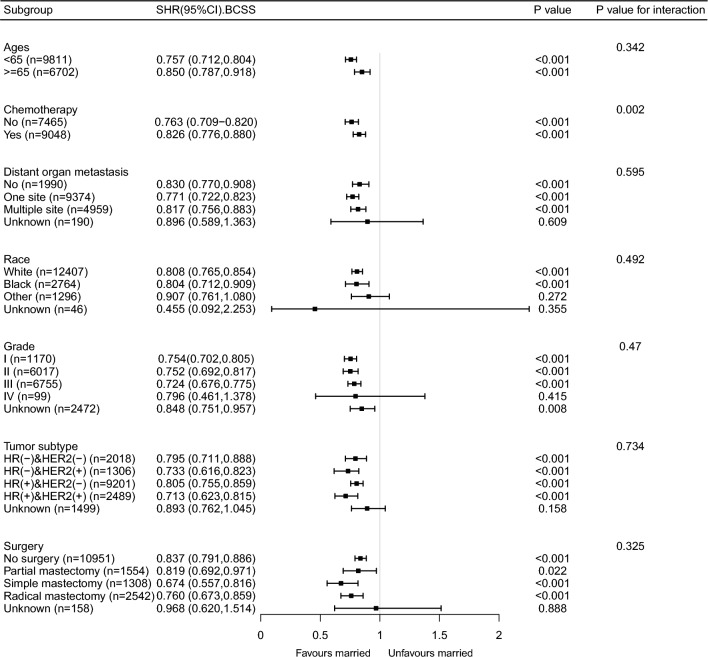
Forest plot of subgroup analysis for breast cancer-specific survival.

**Figure 4:**
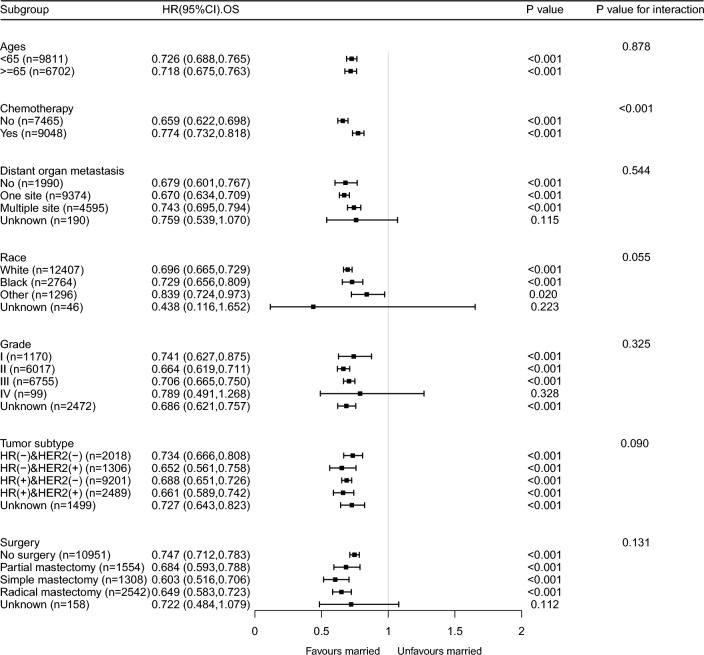
Forest plot of subgroup analysis for breast overall survival.

The results of the sensitivity analysis were consistent with those of the primary analysis (Table 3 and Supplementary Table 1 and Supplementary Fig. 2). The exclusion of patients with missing covariate data (n = 3744) and those with a survival period of less than 1 month (n = 792) after diagnosis did not alter the findings of the study. After adjusting for multiple covariates, marital status remained a significant prognostic factor in patients with MBC. Although several statistically significant interactions were identified in the subgroup analysis, most of them seemed to have little clinical significance (insufficient sample size). Despite this, the positive effect of marital status on BCSS remained consistent among most subgroups, except for grade IV patients (Fig. 3). Similarly, married patients exhibited better OS than unmarried patients in most subgroups, except for grade IV patients (Fig. 4). Furthermore, we examined the effects of marital status on MBC survival in different remote organ metastasis subgroups. The Kaplan–Meier curves of the BCSS and OS rates in different remote organ metastases are shown in Supplementary Fig. 3. Except for the brain metastasis subgroup, the married group had better BCSS and OS than the unmarried group.

### Mediation analysis

**Figure 5:**
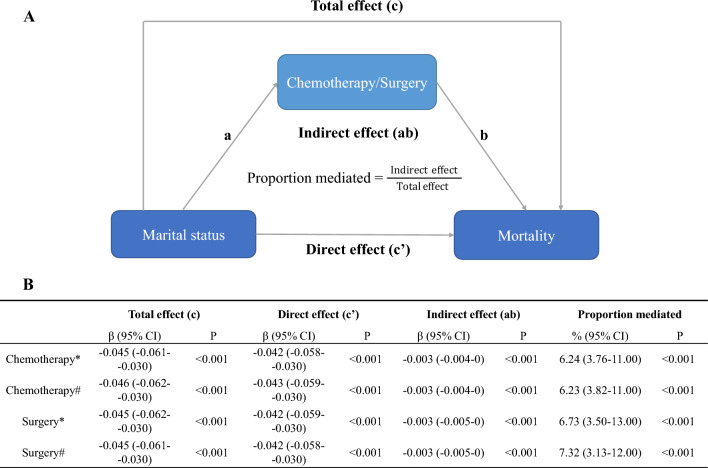
Causal mediation analysis. The total effect of marital status on mortality is represented by path c, whereas the direct effect of marital status on mortality after controlling for the level of chemotherapy/surgery is represented by path c′. The effect of marital status on the level of chemotherapy/surgery is represented by path a; the effect of chemotherapy/surgery on mortality, after controlling for marital status, is represented by path b. The indirect effect of marital status on mortality through chemotherapy/surgery can then be quantified as the product of paths a and b (**A**). Total, direct, and indirect association of marital status with mortality mediated via chemotherapy/surgery (**B**). *Breast cancer-specific mortality; ^#^overall mortality.

We observed that certain factors contribute to greater BCSS and OS rates in married patients, specifically chemotherapy and surgery. Our analysis indicated that the proportion of excess BCSS in married patients compared with unmarried patients was mediated by 6.24% and 6.73% due to chemotherapy and surgery, respectively. Additionally, the proportion of excess OS in married patients relative to that in unmarried patients was mediated by 6.23% and 7.32% due to chemotherapy and surgery, respectively. The indirect effect of marital status on mortality through chemotherapy/surgery can then be quantified as the product of paths a and b (Fig. 5A). Total, direct, and indirect association of marital status with mortality mediated via chemotherapy/surgery (Fig. 5B).

## Discussion

This population-based analysis presents findings that highlight the effect of marital status on survival outcomes in patients with MBC. This study examined a cohort of 16,513 patients with MBC and found that unmarried patients had a significantly higher mortality risk than their married counterparts. After adjusting for demographic variables, clinicopathological tumor characteristics, and treatment strategies, unmarried patients had a 15.5% increased risk of breast cancer-specific mortality and a 19.0% increased risk of overall mortality compared with married patients with MBC. The study concluded that marital status is an independent prognostic factor in patients with MBC. These findings provide further evidence of the importance of social support in the management of MBC and suggest the need for healthcare providers to consider the impact of marital status on treatment decisions and patient outcomes.

Several studies have investigated the association between marital status and breast cancer prognosis^15–17^. Hinyard et al. found that younger married women with breast cancer, particularly those aged 25–34 years, may benefit from additional counseling, psychosocial support, and case management at the time of diagnosis to ensure optimal outcomes^15^. Liu et al. reported that marital status is an independent prognostic factor in patients with inflammatory breast cancer^16^. Zhai et al. divided breast cancer patients into married, divorced/separated/widowed (DSW), and single groups and found that married and single patients had better breast BCSS compared with the DSW patients. However, a subgroup analysis revealed that superior BCSS was only observed in patients older than 35 years, White patients, and patients with ER + /PR + status when comparing single versus DSW^17^. Although previous studies have suggested that marital status is beneficial to the long-term prognosis of patients with breast cancer, a recent systematic review and meta-analysis showed that after population- and age-adjusted estimates in subgroup analysis, the relationship between marital status and breast cancer survival risk is no longer significant^18^. These inconsistent results can be attributed to the heterogeneity of the included population characteristics and the presence of potential confounding factors. Married patients with breast cancer may receive more emotional and financial support, resulting in early diagnosis, effective treatment, and longer OS outcomes. However, whether marriage can provide similar benefits to patients with MBC who are at a high risk of mortality remains unclear^12,13,19^. Therefore, further research is warranted to investigate the relationship between marital status and MBC prognosis.

We included only patients with MBC in our study and controlled for potential confounding factors using multivariable and subgroup analyses. Even after adjusting for multiple factors, marriage remained an independent prognostic risk factor for MBC. However, in the subgroup analysis depicted in Fig. 3, the sample size of grade IV patients was insufficient, as indicated by the wide confidence interval, making it difficult to determine statistical significance. Additionally, chemotherapy and marital status had an interactive effect (p < 0.001) on the survival outcomes of patients with MBC. This finding suggests that marital status may play a more significant role in determining survival outcomes in patients who do not undergo chemotherapy. It is possible that the emotional and social support provided by a spouse or partner in the absence of chemotherapy treatment contributes to improved survival in this subgroup of patients. Breast cancer typically metastasizes to the bone, brain, liver, lungs, and distant lymph nodes, with 70% of breast cancers metastasizing to the bone, nearly 30% to the liver, and 10–30% to the brain^20–22^. The prognosis of patients with MBC with different organ metastases varies, the multiple-site metastases or brain metastases having the poorest survival^23,24^. As only 202 patients with MBC with brain metastasis were identified in our study, and approximately one-third of patients with MBC (n = 4959) had multiple-site metastasis, we used the absence of distant vital organ metastasis as a reference to investigate the increased risk of death with multiple-site metastasis. We analyzed the effects of marital status on the survival of patients with MBC in different subgroups of patients with remote organ metastasis. The Kaplan–Meier curves of BCSS and OS rates in different remote organ metastasis subgroups showed that the BCSS and OS rates of the married group were better than those of the unmarried group, except for brain metastasis (Supplementary Fig. 3). In most cases, brain metastasis is considered a late complication of the disease and occurs after systemic metastases to the lungs, liver, and/or bones, for which few effective treatments are available^25^. Brain metastases are associated not only with poor prognosis but also with neurological impairment, often affecting cognitive and sensory functions^25^. Therefore, the effect of marriage on long-term outcomes in these patients may be overridden, owing to poor outcomes and short survival. The subgroup and sensitivity analyses indicated that the findings were robust.

In this study, three potential mechanisms were examined to explain the relationship between marital status and the survival of patients with MBC. First, married patients with psychological support from their spouses or children may be better able to comply with therapeutic strategies^13,19^. The study also found that married patients are more likely to undergo surgery, chemotherapy, and radiation than unmarried patients with MBC. Through causal mediation analysis, we discovered that surgery and chemotherapy might mediate the association between marital status and survival outcomes in patients with MBC. Second, depression and anxiety in breast cancer patients are related to tumor- and treatment-related inflammatory responses that can induce tumor growth^26,27^. Married patients may receive more social support from their partners and children, which could help them avoid mental illness^28^. Finally, previous studies have shown that married patients have healthier lifestyles, including healthy diet, physical activity, and regular medical checkups, which may be intermediate factors in cancer prevention^7^. Marital status is strongly associated with socioeconomic status. Married patients may receive more emotional and financial support, access to more standardized and complete medical care, and have a better prognosis^11–13^. Future studies should also consider using more comprehensive measures of socioeconomic status, such as education level and occupation, to better elucidate the relationship between socioeconomic status and breast cancer outcomes.

The relationship between marital status and survival is likely to be influenced by various complicated processes. Therefore, studies based on large population databases may be difficult to interpret. This study had some limitations. First, the marital status was recorded only at the time of diagnosis in the SEER database. However, marital status is a dynamic variable that may change over time and affect the outcomes. Additionally, the quality of marriage was not recorded, which could have affected the level of support available to the patient. Second, our retrospective study was limited in its ability to define the causal relationship between marital status and survival. Third, the SEER database lacks socioeconomic and educational information, which may have influenced the results. Fourth, the database did not record histories of anxiety, depression, or other psychological illnesses in patients with MBC. Therefore, we were unable to evaluate the baseline mental condition of the patients at the time of diagnosis. Finally, the omission of certain variables, such as surgery, radiotherapy, chemotherapy, and molecular subtypes after 2015, may have limited the generalizability of our findings to more recent periods, although significant developments have been achieved in breast cancer treatment and management over the past few years.

Nonetheless, our findings suggest that marital status has a significant effect on the survival of patients with MBC, emphasizing the substantial and consistent effect of marriage. Our results imply that social support programs targeting vulnerable populations, notably unmarried individuals, are likely to significantly enhance the likelihood of recovery. These interventions could be cost-effective in improving the therapeutic outcomes among unmarried individuals with cancer. In particular, SEER provides a large and diverse population-based sample with long-term follow-up, allowing the investigation of rare outcomes and evaluation of changes in cancer care over time. SEER also provides detailed clinical and pathological information that enables the exploration of various factors related to breast cancer outcomes.

## Conclusion

We found that marital status was an independent prognostic indicator of MBC. The survival advantage of married patients was greater than that of unmarried patients in terms of BCSS and OS. The findings of this study warrant further investigation.

## Supplementary Information

Supplementary Information.

## Data Availability

The data analyzed in this study are available through the SEER database (https://seer.cancer.gov/). We completed the SEER project registration and obtained authorization for data extraction (reference number: 18124-Nov 2020).
