# Supplementary material for: Association between marital status and all-cause mortality of patients with metastatic breast cancer: a population-based study
Source: Sci Rep. 2023 Jun 5;13:9067. doi: 10.1038/s41598-023-36139-8 (PMC10241782; doi:10.1038/s41598-023-36139-8)
Supplement: Supplementary file 1 — Supplementary Information. [file 41598_2023_36139_MOESM1_ESM.pdf]

Association of marital status and all-cause mortality of metastatic breast cancer patients: A population-based study

Supplementary materials

Table 1 Gray’s test and Fine–Gray proportional subdistribution hazard method analysis of breast cancer-specific survival (BCSS) for patients with metastatic breast cancer, and sensitivity analysis.

Figure. S1 Percentage of missing variables.

Figure. S2 Kaplan-Meier survival curves of overall survival(A) and breast cancer-specific survival(B) on sensitivity analysis. We excluded all patients with missing covariable data (n=3744) and those who survived less than 1 month (n=792) after diagnosis. The final sample size for inclusion in the sensitivity analysis was 11977 patients, 5616 married and 6361 unmarried patients.

Figure. S3 Kaplan-Meier survival curves of breast cancer-specific survival (BCSS) in different distant organ metastasis subgroup (A: without distant organ metastasis; B: bone metastasis; C: liver metastasis; D: brain metastasis; E: lung metastasis; F: multiple organ metastasis) and overall survival (OS) in different distant organ metastasis subgroup (G: without distant organ metastasis; H: bone metastasis; I: liver metastasis; J: brain metastasis; K: lung metastasis; L:multiple organ metastasis) MBC patients according to marital status.

Table S1 Gray's test and Fine–Gray proportional subdistribution hazard method analysis of breast cancer-specific survival (BCSS) for patients with metastatic breast cancer, and sensitivity analysis.

**Characteristic (n)**	**Univariable analysis**		**Multivariable analysis**	
	**Gray's test**	***p*-value**	**SHR (95% CI)**	***p*-value**
**BCSS**				
**Marital status**	118.579	<0.001		<0.001
Unmarried (8949)			Reference	
Married (7564)			0.840 (0.799-0.883)	
**Ages**	12.086	0.001		0.845
<65 (9811)			Reference	
>=65 (6702)			1.005 (0.954-1.060)	
**Race**	75.228	<0.001		<0.001
White (12407)			Reference	
Black (2764)			1.168 (1.095-1.245)	<0.001
Other (1296)			1.016 (0.929-1.112)	0.727
Unknown (46)			0.500 (0.245-1.022)	0.057
**Grade**	262.687	<0.001		<0.001
I (1170)			Reference	
II (6017)			1.331 (1.194-1.483)	<0.001
III (6755)			1.809 (1.621-2.020)	<0.001
IV (99)			2.240 (1.685-2.979)	<0.001
Unknown (2472)			1.499 (1.330-1.669)	<0.001
**Subtype**	622.276	<0.001		<0.001
HR(-) & HER2(-) (2018)			Reference	
HR(-) & HER2(+) (1306)			0.500 (0.448-0.558)	<0.001
HR(+) & HER2(-) (9201)			0.496 (0.458-0.536)	<0.001
HR(+) & HER2(+) (2489)			0.418 (0.381-0.458)	<0.001

Unknown (1499)			0.565 (0.506-0.630)	<0.001
**Chemotherapy**	76.829	<0.001		<0.001
No (7465)			Reference	
Yes (9048)			0.845 (0.798-0.894)	
**Radiation**	18.592	<0.001		NI
No (10937)				
Yes (5207)				
Unknown (369)				
**Surgery**	462.431	<0.001		<0.001
No surgery (10951)			Reference	
Partial mastectomy (1554)			0.629 (0.575-0.688)	<0.001
Simple mastectomy (1308)			0.581 (0.526-0.642)	<0.001
Radical mastectomy (2542)			0.734 (0.685-0.785)	<0.001
Unknown (158)			1.021 (0.823-1.266)	0.850
**Distant organ metastasis**	702.044	<0.001		<0.001
No (1990)			Reference	
One site (9374)			1.246 (1.147-1.354)	<0.001
Multiple sites (4959)			1.937 (1.774-2.115)	<0.001
Unknown (190)			1.393 (1.109-1.748)	0.004

Note: Distant organ metastasis only included bone, liver, lung, and brain metastases in the SEER database (2010-2015). NI: not included in the multivariable survival analysis. SHR: subdistribution hazard ratio. Patients with an unknown cause of death were classified as dying of tumor-related-cause. There were 7484 deaths from tumors, 2519 deaths from other causes, and 6,510 survivors at the follow-up endpoint.

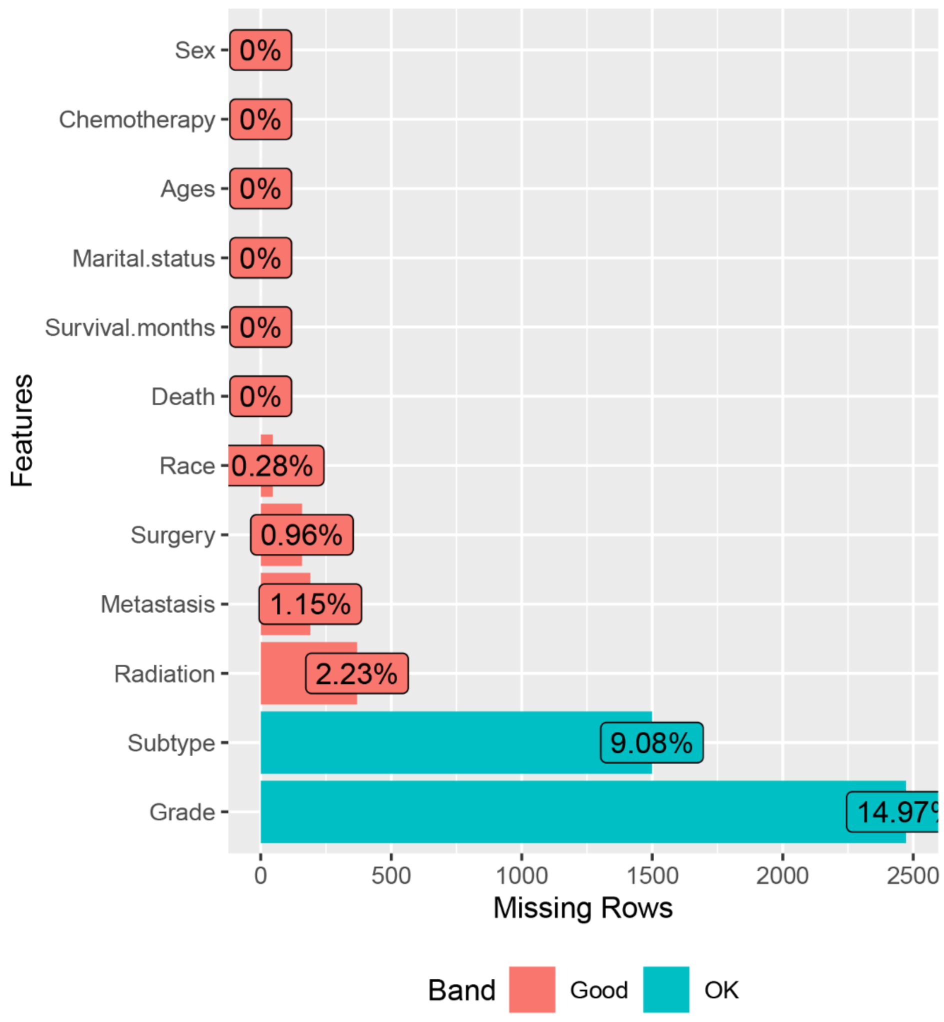

Figure. S1 Percentage of missing variables.

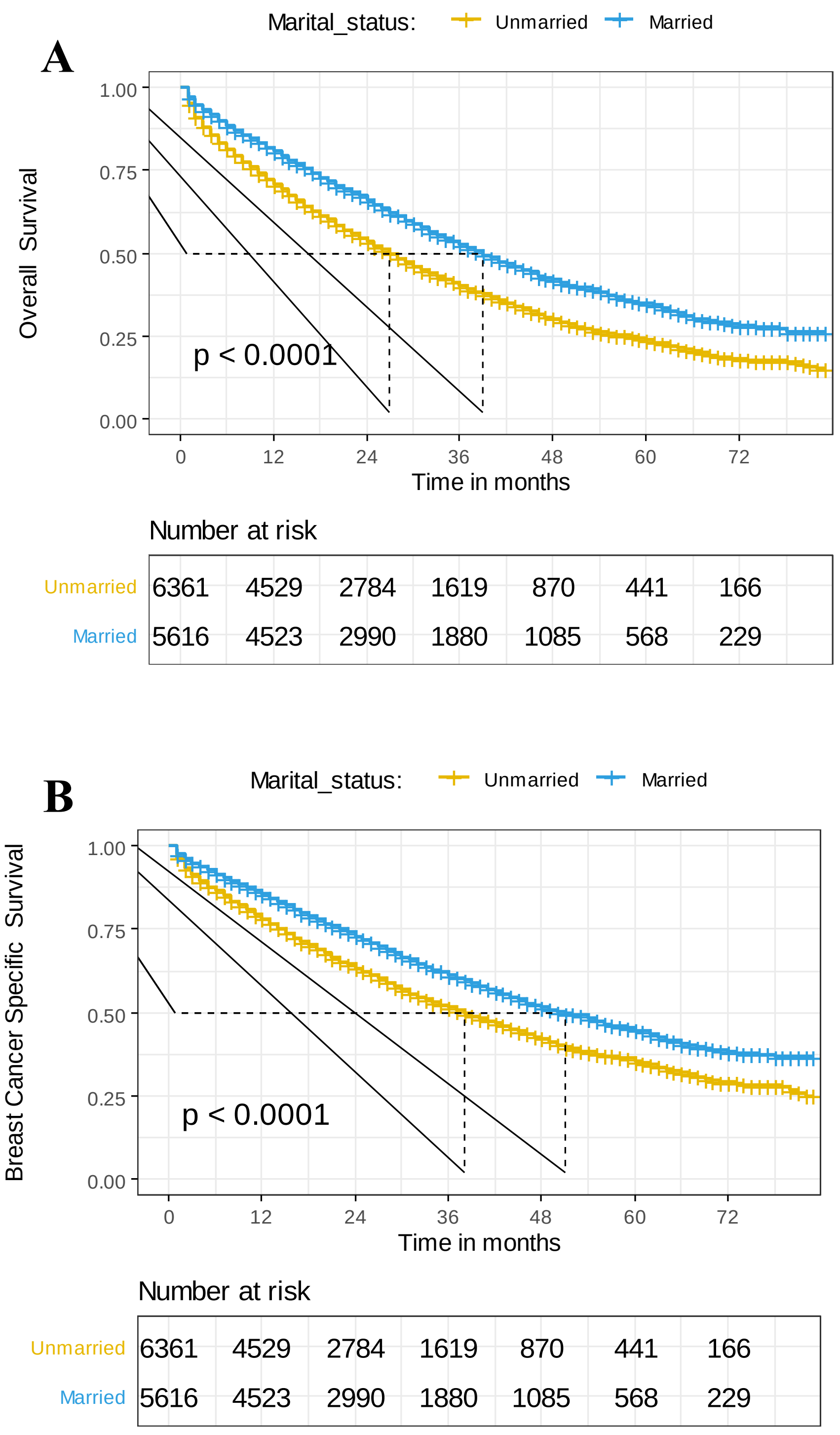

Figure. S2 Kaplan-Meier survival curves of overall survival(A) and breast cancer-specific survival(B) on sensitivity analysis. We excluded all patients with missing covariable data (n=3744) and those who survived less than 1 month (n=792) after diagnosis. The final sample size for inclusion in the sensitivity analysis was 11977 patients, 5616 married and 6361 unmarried patients.

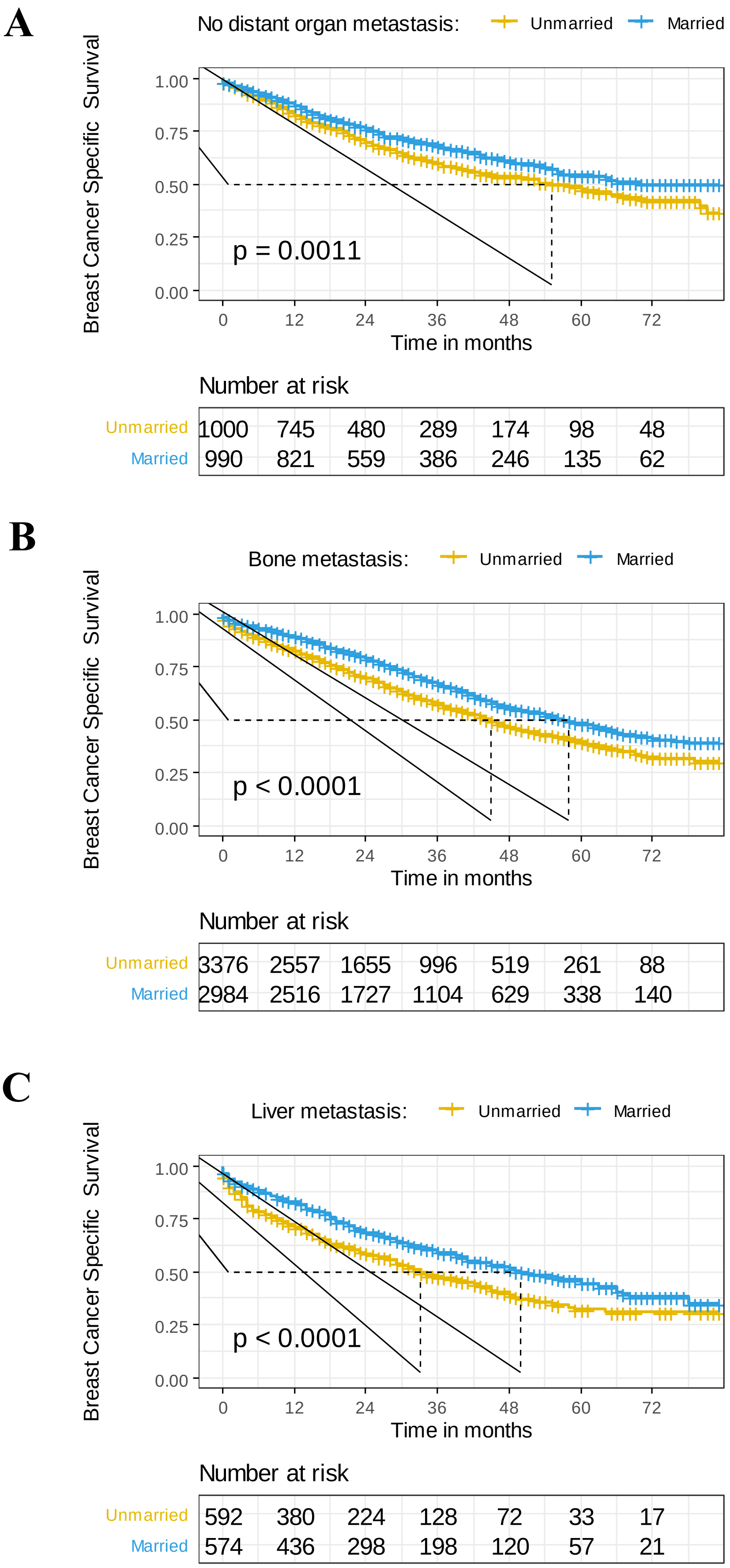
A
No distant organ metastasis:
Unmarried
Married
Breast Cancer Specific Survival
1.00
0.75
0.50
0.25
0.00
p = 0.0011
0
12
24
36
48
60
72
Time in months
Number at risk
Unmarried 1000 745 480 289 174 98 48
Married 990 821 559 386 246 135 62
B
Bone metastasis:
Unmarried
Married
Breast Cancer Specific Survival
1.00
0.75
0.50
0.25
0.00
p < 0.0001
0
12
24
36
48
60
72
Time in months
Number at risk
Unmarried 3376 2557 1655 996 519 261 88
Married 2984 2516 1727 1104 629 338 140
C
Liver metastasis:
Unmarried
Married
Breast Cancer Specific Survival
1.00
0.75
0.50
0.25
0.00
p < 0.0001
0
12
24
36
48
60
72
Time in months
Number at risk
Unmarried 592 380 224 128 72 33 17
Married 574 436 298 198 120 57 21

D

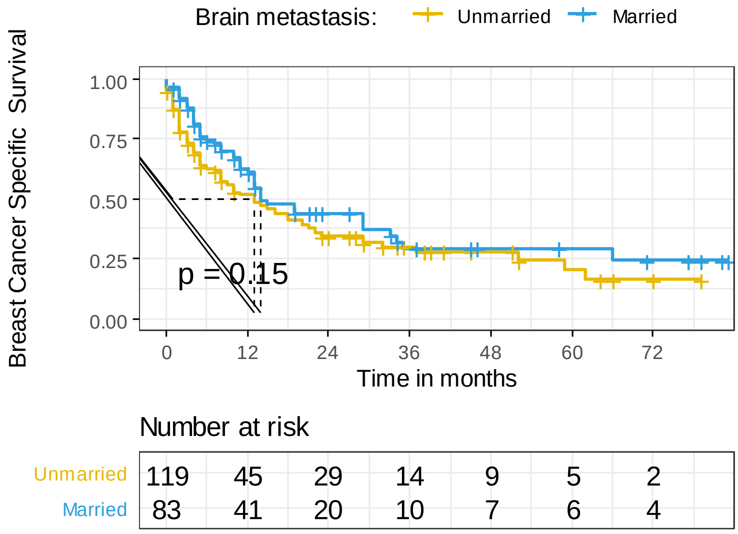

Brain metastasis:
Unmarried
Married
Breast Cancer Specific Survival
1.00
0.75
0.50
0.25
0.00
p = 0.15
0
12
24
36
48
60
72
Time in months
Number at risk
Unmarried 119 45 29 14 9 5 2
Married 83 41 20 10 7 6 4

E

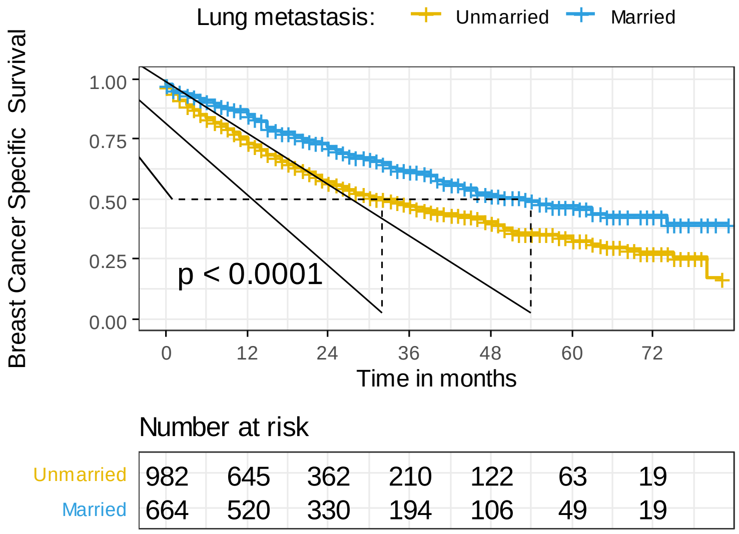

Lung metastasis:
Unmarried
Married
Breast Cancer Specific Survival
1.00
0.75
0.50
0.25
0.00
p < 0.0001
0
12
24
36
48
60
72
Time in months
Number at risk
Unmarried 982 645 362 210 122 63 19
Married 664 520 330 194 106 49 19

F

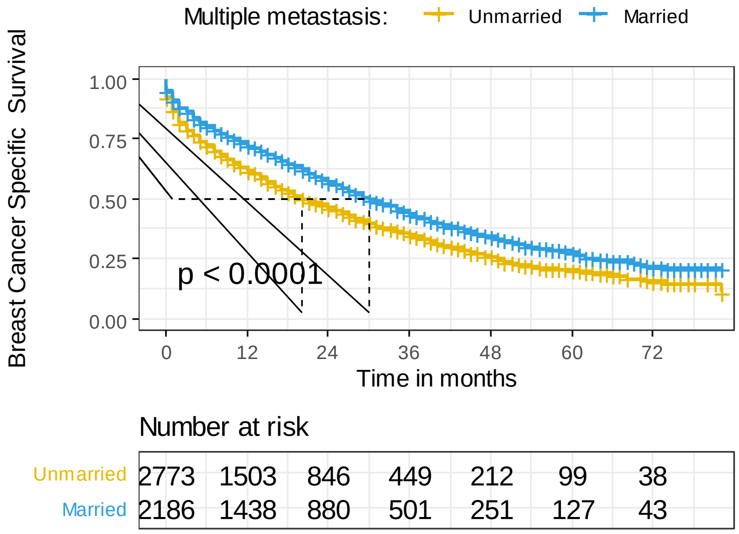

Multiple metastasis:
Unmarried
Married
Breast Cancer Specific Survival
1.00
0.75
0.50
0.25
0.00
p < 0.0001
0
12
24
36
48
60
72
Time in months
Number at risk
Unmarried 2773 1503 846 449 212 99 38
Married 2186 1438 880 501 251 127 43

G

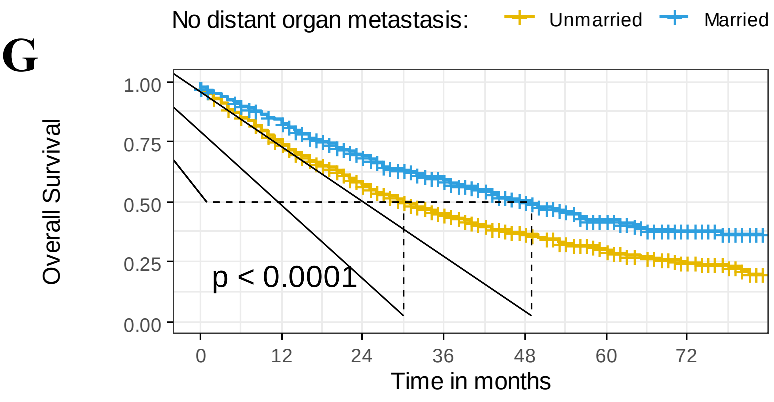

Number at risk

Unmarried	1000	745	480	289	174	98	48
Married	990	821	559	386	246	135	62

H

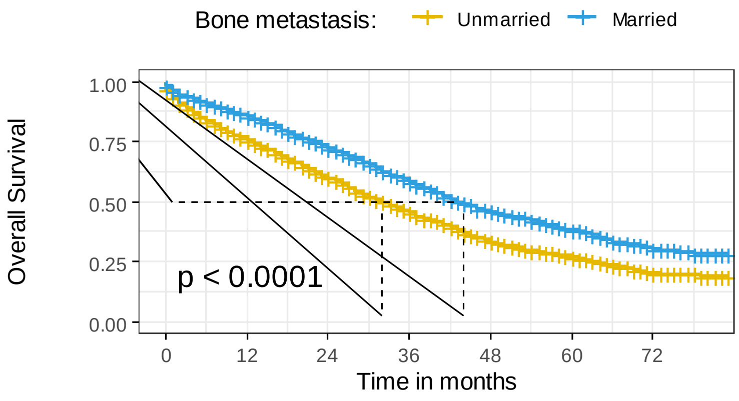

Number at risk

Unmarried	3376	2557	1655	996	519	261	88
Married	2984	2516	1727	1104	629	338	140

I

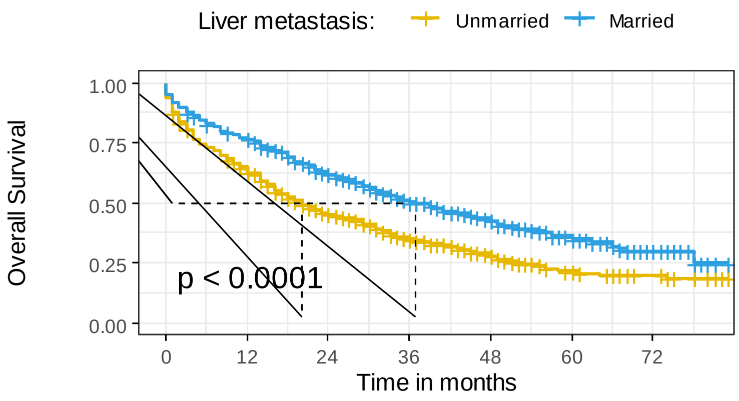

Number at risk

Unmarried	592	380	224	128	72	33	17
Married	574	436	298	198	120	57	21

J

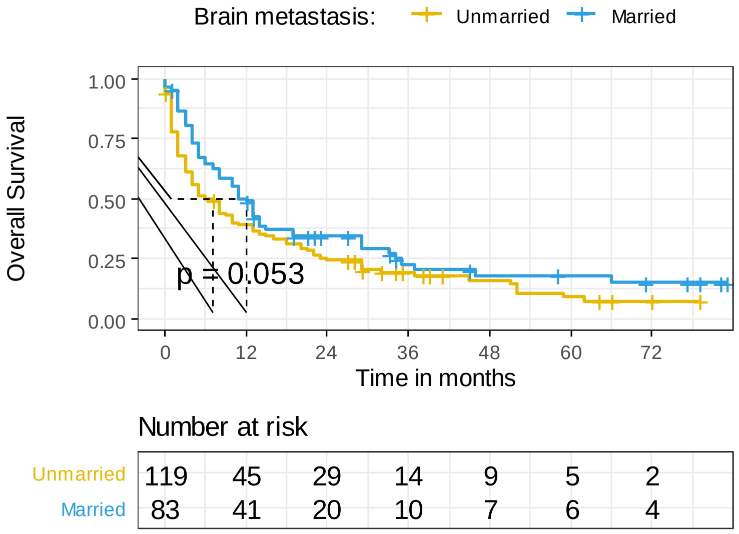
Brain metastasis:
Unmarried
Married
Overall Survival
1.00
0.75
0.50
0.25
0.00
p = 0.053
0
12
24
36
48
60
72
Time in months
Number at risk
Unmarried 119 45 29 14 9 5 2
Married 83 41 20 10 7 6 4

K

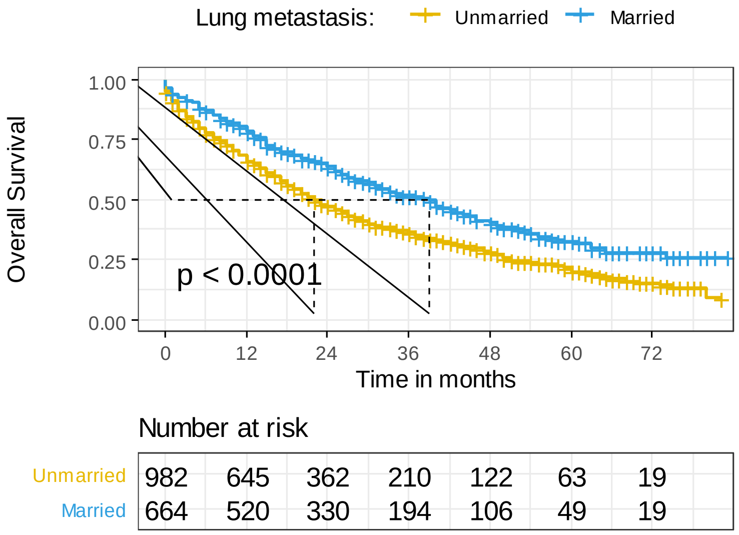
Lung metastasis:
Unmarried
Married
Overall Survival
1.00
0.75
0.50
0.25
0.00
p < 0.0001
0
12
24
36
48
60
72
Time in months
Number at risk
Unmarried 982 645 362 210 122 63 19
Married 664 520 330 194 106 49 19

L

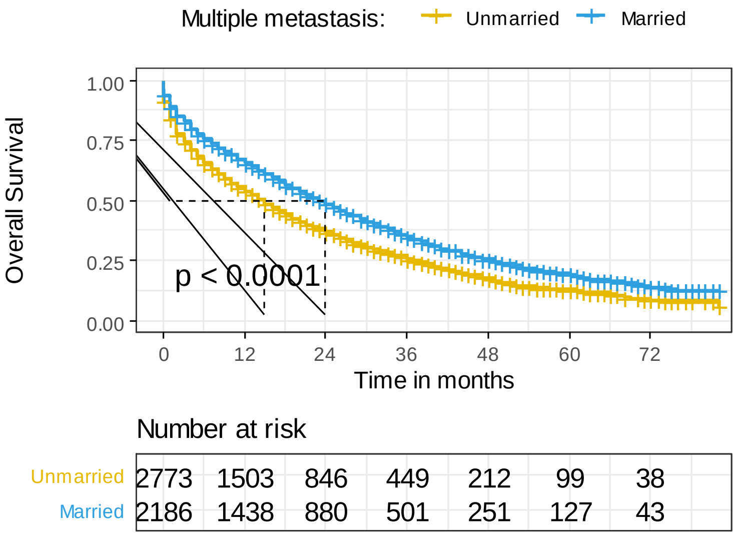
Multiple metastasis:
Unmarried
Married
Overall Survival
1.00
0.75
0.50
0.25
0.00
p < 0.0001
0
12
24
36
48
60
72
Time in months
Number at risk
Unmarried 2773 1503 846 449 212 99 38
Married 2186 1438 880 501 251 127 43

Figure. S3 Kaplan-Meier survival curves of breast cancer-specific survival (BCSS) in different distant organ metastasis subgroup (A: without distant organ metastasis; B: bone metastasis; C: liver metastasis; D: brain metastasis; E: lung metastasis; F: multiple organ metastasis) and overall survival (OS) in different distant organ metastasis subgroup (G: without distant organ metastasis; H: bone metastasis; I: liver metastasis; J: brain metastasis; K: lung metastasis; L:multiple organ metastasis) MBC patients according to marital status.
